# Meeting of the Northern Ohio Dental Society

**Published:** 1872-08

**Authors:** 


### PROCEEDINGS OF SOCIETIES.

### Meeting of the Northern Ohio Dental Society.

PUT-IN BAY ISLAND,
May 28, 1872, 10, A. M.

Association called to order by President Dr. Waye.

MEMBERS PRESENT.

D. R. Jennings, C. Buffett, L. Buffett, J. E. Robinson, J. Stephan, Cleveland; E. J. Waye, H. M. Reid, Sandusky; C. B. Knowlton, Elyria; H. Terry, Norwalk; A. F. Price, Fremont; B. T. Spelman, Detroit; C. H. Harroun, Toledo; J. Taft, Cincinnati, and several visitors.

Minutes of last meeting read and approved.

Treasurer's report received and referred to Auditing Committee.

Dr. Knowlton, appointed Auditor.

Report of Executive Committee received and accepted.

Drs. J. E. Robinson and C. Buffett were appointed to fill vacancies in Examining Committee.

Dentists present, not members of the Association, were invited to participate in the discussions.

The Auditing Committee reported the Treasurer's account correct.

Motion made and carried that the resolution offered at the previous meeting to change Article 9, of the Constitution, bs reconsidered and amended, so as to read second Tuesday of June, instead of last Tuesday of May. Adjourned to 2 o'clock P. M.

Meeting called to order by the President. Minutes of morning session read and approved.

DISCUSSIONS.

1st. In what cases should the dental pulp be destroyed. and best methods of preserving the same?

Dr. Spelman still does, and thinks he always shall at times destroy pulps; yet the Profession's attention should be directed to the saving of them—the nerve once destroyed, the vitality of the tooth is gone, its structure becomes changed. The construction of the dental pulp is a peculiar one; a very little congestion, or even determination, causes severe trouble. The small foramin through which the nerve arteries, veins and connective tissue pass, prevents our treatment being as successful as in other parts. In filling over exposed pulps, or those nearly so, must first restore the tissue to a normal condition, and then use some non-conducting material. We sometimes expose a small portion of nerve in excavating, when there has been no previous trouble; we here have no congestion, but a fresh wounded tissue and determination will soon set in, followed by congestion; this we must allay and fill so that thermal changes will not affect the nerve. To destroy the nerve, uses an instrument on the heroic plan; if any remedy, uses carbolic acid, crystals inserted in the cavity, and then drilled.

Dr. Price uses arsenic to destroy nerves. Cannot always succeed with carbolic acid. Would save the life of the nerve if possible.

Dr. Evans feels some doubt as to saving the teeth when the nerve has been destroyed; caps with oxychloride of zinc, after applying a coating of collodian, so as to form a thin film-has had but few failures.

Dr. Knowlton—There is no organ that passes so soon through the stage of irritation, inflamation and ulceration, as the dental pulp, which soon dies of strangulation at foramin.

Dr. Robinson believes too many nerves are destroyed; can generally save when the nerve is only in an irritated condition, but not congested.

Dr. Spelman cited a case in which he removed the body of the pulp—leaving the nerve in the roots—put in a temporary filling, which upon removing some time after, found the nerve in a healthy condition.

Dr. Jennings believes oxychloride of zinc is not a fit filling to be applied directly to the nerve; would first use Hill's stopping as a capping; fills roots with oxychloride.

Dr. L. Buffett—When the nerve is in a congested state, it cannot be saved; does not favor the oxychloride of zinc applied directly to nerve, it acts as an irritant; would use some non-conductor before putting in the oxychloride; condemns the too free use of carbolic acid, and also of arsenic.

Dr. Knowlton uses rubber, with bisulphide of carbon as capping, to exposed pulps.

Dr. C. Buffett believed that oxychloride of zinc could not be applied to an exposed nerve without destroying it; would first use collodian.

Dr. Reid had filled childrens' teeth by using collodian and oxychloride of zinc.

Dr. Knapp does not believe that the nerves are saved, alive, after they are once exposed; but are shriveled up, and would sometimes ulcerate, but rarely gave trouble.

Dr. Stephan—Can see no reason why nerves should not be saved if we use some non-conducting material that would not press upon it.

Dr. Stroud, some two years ago, thought there was no use in destroying nerves, but had many failures when he tried to save all.

Dr. Waye—Many good men claim they can save nerves or even parts of nerves, in almost any stage of disease; for himself had not so much confidence; uses collodian and Hill's stopping when there was slight exposure; thought irritation was likely to follow the use of any escharotic, when there was a thin lamina of bone over the nerve.

Committee on Membership presented the names of C. T. Stroud and G. G. Ashcraft, of Sandusky, who were elected members. Adjourned to 8 P. M.

EVENING SESSION, 8 o'clock, P. M.

Minutes read and approved. Committee on Membership presented the name of D. F. Wemple, of Sandusky, who was elected a member. *Discussions resumed.*

Dr. J. Taft regarded the treatment and preservation of exposed pulps to be of the greatest importance.

Under proper treatment they may be restored to health, and their vitality retained, much more frequently than is generally allowed.

The vitality of the dentine depends almost wholly upon the pulp; and living dentine is far more resistant to decay producing agents, than the dead. Periostial trouble is of rare occurrence till after the pulp is destroyed; this alone should be a great incentive to its preservation.

The pulp usually becomes affected after its exposure, but sometimes while there is still a considerable covering of dentine, because of its susceptibility to irritation, and the ready transmission which the dentine sometimes gives to pernicious influences, such as irritating agents operating upon the dentine, excessive thermal changes, and systemic predisposing conditions.

If we practically ignore physiology and pathology, we must expect failures—disease may be ready to break out in the system, and we not perceive it.

The proper treatment of the pulp consists in placing it back in its natural condition, allowing no space in its chamber—using some non-conductor. Collodion, or gutta percha dissolved in chloroform is the best. On this fills with oxychloride of zinc, which let harden, then cut out enough to give the cavity proper shape, and fill with gold.

As to the treatment of pulps after they become diseased, those that become much diseased we perhaps can not always save; others that are not much diseased, though fully exposed, can be successfully treated and saved. After pulps have pus in their chamber they can then sometimes be saved; when the pulp must be taken out, the tissue in the root can be often saved; when this can be done periostial inflammation is less likely to occur. The character of exudation from the pulp is not always the same. The covering should be something that would not be effected by any of the secretions. Different sections of the country will bring different influences. Various malarial diseases will most certainly affect the severing of the pulps of the teeth.

On motion Dr. C. R. Taft, of Mansfield, was elected an honorary member of the association.

Adjourned to 9 o'clock A. M.

WEDNESDAY MORNING, 9 A. M.

Committee on Membership reported the name of S. P. Hildreth, of Norwalk, as an active member of this association. C. C. Jenkins, of Ann Arbor, and Knapp, of Adrian, Michigan, as honorary members. All were elected. The association then proceeded to the election of officers, resulting as follows:

President—H. Terry; Vice President—B. F. Robinson; Recording Secretary—A. F. Price; Corresponding Secretary—L. Buffett; Treasurer—J. E. Robinson.

Examining Committe—R. Jennings, C. Buffett, C. B. Knowlton.

Delegates to American Dental Association—C. R. Butler, B. T. Spelman, A. F. Price, E. J. Waye, R. Jennings, J. F. Siddall, C. B. Knowlton. Dr. H. Terry, the President Elect, was then conducted to the chair.

The retiring President, Dr. E. J. Waye, then delivered a very interesting and able address, complimenting the profession upon what had been done in the past, and urged upon them greater exertion in the future.

The next subject for discussion was Diseases and Treatment of the Alveolus.

Dr. Spelman—Every effort should be made to subdue the inflammation of the nerve of a tooth before there is pus formed, resulting in alveolar abscess. If the periostium is once destroyed it can never be restored again.

Dr. J. Taft a new periostium will be formed if the surrounding parts became healthy—had seen cases where the teeth were loose and abscessed; having restored the soft parts to a healthy condition new alveolus was formed and the teeth became firm. In the inflammatory condition of the alveolus, would advise the same general treatment as for inflammation elsewhere.

Salivary calculus would cause absorption of the alveolus with or without the loss of the soft tissues; sometimes extended so as to occasion necrosed alveolus—the grade of vitality having much to do with the extent of the disease.

Would begin treatment by first removing the cause when there is necrosed bone, cut away as much as possible, and inject the elixir of vitrol, so as to clear away and dissolve any remaining portion of dead bone. The living tissue will not be materially affected by it at least till the dead is destroyed; it is seldom necessary to make more than one application.

In treating a tooth for alveolar abscess, would thoroughly cleanse the nerve canal, break up the sac if possible, and introduce some escharotic—preferred carbolic acid.

In some individuals the systemic condition was such that it was almost impossible to cure alveolar abscess by any local treatment. Malarious districts were detrimental to their cure.

Adjourned to 2 P. M.

2 O'CLOCK P. M.

Meeting called to order; minutes read and approved. Drs. Fields and Thomas of Detroit, were introduced, and invited to take part in the discussions. The subject of the morning discussions was then resumed.

Dr. C. R. Taft thought new periostium could not be formed after a tooth had lost its vitality from death of the nerve.

Dr. Spelman thought that the periosteum was not a bone producing tissue.

Dr. J. Taft—It makes no difference whether the periosteum be a bone producing tissue or not, it can be restored, even when partially destroyed, after the death of the nerve—would advise the profession to experiment more with the elixir of vitrol.

Dr. Knapp's treatment of alveolar abscess was to clean out the nerve canal, inject carbolic acid and fill the root, cut through the process and break up the sac.

Dr. Field preferred treating through nerve canal and not filling until abscess was cured.

Dr. Harroun would treat through nerve canal if there was sufficient opening through the end of the root; if not, fills root and treats through process; related a case of alveolar abscess where nerve of the tooth was alive.

Dr. Spelman first cleanses the canal and then fills with gold, cuts through the process to apex of the root and breaks up the sac; could generally control his patients so they would stand the heroic treatment; had successfully treated a good many cases of alveolar abscess by simply cleaning and filling nerve canal; found more trouble in treating lower molars than any of the other teeth, they were more liable to form abscesses after the nerve was dead; would advise all dentists to use all the skill in their power to save the nerve alive in those teeth.

Dr. J. Taft, in treating lower molars should wash out the pulp canal thoroughly and frequently; makes a large opening through process and breaks up the sac as thoroughly as possible; when there is a roughened condition of the root, from deposits of salivary calculus or other causes, clean off as much as possible and inject elixir of vitrol.

Adjourned to 8 o'clock P. M.

EVENING SESSION.

Meeting called to order. Subject of discussion, Artificial Dentures.

Dr. Waye had used celluloid; did not succeed very well at first, but later it had given good satisfaction.

Dr. Harroun would use plain teeth when he used the celluloid base; thought it more liable to shrink from the pins when block teeth were used; considered the continuous gum superior to all other bases.

Dr. Waye preferred gold plate with rubber attachment for durability.

Dr. Harroun for cheap work made silver plate, electroplated with gold, and teeth fastened by rubber.

Dr. Knapp preferred aluminum with rubber attachments for a cheap base.

Dr. Fields considers the continuous gum the *ne plus ultra* of all bases; it is cleaner, more durable, and gives a dentist a better opportunity of displaying his artistic skill in arranging the teeth.

Dr. J. Taft did not think any particular material was applicable to every case; plates that could be worn in some mouths with impunity, would not be tolerated in others. The objects of artificial dentures are to restore as far as possible the functions of the natural organs, but most of the artificial dentures fall far short of this; few are able to masticate their food properly, or have their voice restored to its natural condition; great care should be taken in making artificial dentures to restore all that has been lost by the absence of the natural organs. We should spend more time in trimming and forming the trial plate, until we get a natural expression to the face; in selecting teeth should select those of a proper color to correspond with the age and complexion of the patient, and of proper shape to correspond with the features, arranged so as to most thoroughly accomplish the purposes of mastification; do not think platina or gold injurious to the mucous membrane; think more than one-half the rubber plates worn are injurious. Rubber, silver and celluloid might be worn by some, but in a large number of cases would be injurious. Would urge upon the profession more thorough study and education in regard to this particular branch; when the people are given to understand the poor substitutes given for the natural organs, they will take more pains to save them.

Dr. Hildreth—A dentist to be a successful plate workman, must be an artist of the highest order, and judging from what he had seen, thought there were not a very large proportion of them possessed with that qualification.

Adjourned to meet at 10 A. M.

THIRD DAY.

Morning chiefly devoted to clinics, Dr. Fields operating, using Leslie's crystaline gold.

On motion Dr. Geo. Fields and G. R. Thomas, of Detroit, were made honorary members of the association.

Adjourned to meet at Put-in-Bay, the second Tuesday of June, 1873.

